# *Zingiber officinale* Improves Cognitive Function of the Middle-Aged Healthy Women

**DOI:** 10.1155/2012/383062

**Published:** 2011-12-22

**Authors:** Naritsara Saenghong, Jintanaporn Wattanathorn, Supaporn Muchimapura, Terdthai Tongun, Nawanant Piyavhatkul, Chuleratana Banchonglikitkul, Tanwarat Kajsongkram

**Affiliations:** ^1^Neuroscience Program and Graduate School, Faculty of Medicine, Khon Kaen University, Khon Kaen 40002, Thailand; ^2^Neuroscience Laboratory Unit, Department of Physiology, Faculty of Medicine, Khon Kaen University, Khon Kaen 40002, Thailand; ^3^Department of Psychiatry, Faculty of Medicine, Khon Kaen University, Khon Kaen 40002, Thailand; ^4^Department of Pharmaceutical and Natural Products, Thailand Institute of Scientific and Technological Research, Pathumthani 12120, Thailand

## Abstract

The development of cognitive enhancers from plants possessing antioxidants has gained much attention due to the role of oxidative stress-induced cognitive impairment. Thus, this study aimed to determine the effect of ginger extract, or *Zingiber officinale*, on the cognitive function of middle-aged, healthy women. Sixty participants were randomly assigned to receive a placebo or standardized plant extract at doses of 400 and 800 mg once daily for 2 months. They were evaluated for working memory and cognitive function using computerized battery tests and the auditory oddball paradigm of event-related potentials at three different time periods: before receiving the intervention, one month, and two months. We found that the ginger-treated groups had significantly decreased P300 latencies, increased N100 and P300 amplitudes, and exhibited enhanced working memory. Therefore, ginger is a potential cognitive enhancer for middle-aged women.

## 1. Introduction

Recent findings suggest that middle-aged women usually develop some form of cognitive impairment. It was found that middle-aged women performed poorly in various areas of cognitive function including attention, calculation and immediate recall (assessed using Minimental state examination (MMSE)) [1]. Evidence has also shown that oxidative stress contributes to cognitive impairment as age advanced [2]. Due to the increase in the middle-aged population, an abundance of research has focused on the development of cognitive enhancers from medicinal plants reputed for antioxidant and cognitive enhancing effects. Ginger, or *Zingiber officinale*, a plant in the family of *Zingiberaceae*, has longterm been used as both a spice and as a medicine in Asian, Indian, and Arabian folklore. The rhizomes of *Zingiber officinale *exhibit a wide range of pharmacological properties including antilipidemia [3], antiemetic [4], anti-inflammation, and antiarthritis [5]. According to Arabian folklore, ginger has been claimed to improve memory. Moreover, it has also been traditionally used as an ingredient for cognitive enhancement. Our preliminary data in Wistar rats showed that ginger rhizomes extract could enhance memory and protect against brain damage [6]. In addition, it was also reported to have antioxidant effects [7, 8]. Based on the antioxidant and cognitive enhancing effects of ginger rhizomes extract, the present neuropsychological and electrophysiological study aims to determine the effect of ginger rhizomes extract on the cognitive function of middle-aged women.

## 2. Materials and Methods

### 2.1. Participants

Sixty healthy, Thai, middle-aged women (mean age 53.40 ± 3.57 years) were recruited to participate in the present study, which was approved by the Ethical Committee of the Faculty of Medicine at Khon Kaen University. Prior to investigation, each volunteer provided informed consent and completed the medical health questionnaire. Participants were also screened for physical health by a physician in order to assure healthy condition. Inclusion criteria were healthy, middle-aged, Thai National women between the ages of 50 and 60 residing in the Northeast Region of Thailand. Exclusion criteria included any history of cardiovascular disease, respiratory disease, neuropsychological disease, head injury, diabetes, cancer, alcohol addiction, and anyone who smoked more than 10 cigarettes per day because all mentioned conditions could produce the disturbance of cognitive function. Individuals taking prescribed, nonprescribed drugs, or nutraceutical compounds known to influence the function of the nervous system were excluded. Participants were randomly divided into 3 separate groups: placebo, *Zingiber officinale*  400 mg, and *Zingiber officinale* 800 mg.

### 2.2. The Preparation of the Standardized Extract of *Zingiber officinale*

A standardized extract of *Zingiber officinale *was prepared by the Thailand Institute of Scientific and Technological Research in Pathum Sthani, Thailand. Standardization and conformity of the extract were assured by strict in-process controls during manufacture and complete analytical control of the resulting dry extract. In brief, the dried ginger rhizome powder was extracted with 95% ethanol in a stainless steel tank for 5 to 10 days. The filtrate was evaporated to dryness under a vacuum at 35°C on a rotary evaporator. The production yield of the extract was 6.84% w/w. The phenolic compound of standardized ginger extract contained 7.33% w/w of 6-gingerol and 1.34% w/w of 6-shogaol.

### 2.3. Procedures and Treatments

This study was 2 months in duration and was double blind, placebo controlled, and arranged with randomized trials. A random list of numbers was computer-generated. After being randomly assigned to treatment groups, each participant received one capsule of either the placebo or ginger extract (400 or 800 mg) once daily. The selected doses of *Z. officinale* are based on the dosage range that produces cognitive enhancing effect in animal model and the safety range. The placebo and ginger capsules had the same color, texture, size, and odor. All participants were screened for baseline intellectual function using standard progressive matrices (SPMs) in order to avoid confounding error induced by the intellectual function problem. Participants were assessed for cognitive performance after 1 and 2 months of treatment. According to the evaluation, all experimenters and staff were instructed to follow a strict protocol and were told not to discuss any issues related to the use of medication. The medication compliance was monitored by interview and counting the remaining medication, and the side effect was assessed via interview, self-report, and physical exam in each visit. Subjects were requested to call the study center if they experienced any medical problems during the 60 days of study duration. At the end of the study, they were also asked about any adverse experiences.

### 2.4. Event-Related Potentials (ERPs)

#### 2.4.1. ERP Recording

All subjects were assessed for cognitive performance using the classic “oddball paradigm” of auditory event-related potentials (N100 and P300 amplitudes and latencies) [9]. The electroencephalogram (EEG) was recorded via Cz, and linked mastoids were used as reference for the electrode. The resistance of the electrodes was kept below 5 kohm. The analog filter band pass was 1–100 Hz [10]. For each stimulus, an epoch of 500 ms duration including a 100 ms prestimulus period was extracted from the continuous EEG. Epochs with a voltage change below 0.1 *μ*V or above 70 *μ*V were rejected from further analysis.

#### 2.4.2. ERP Measurement

The subjects listened to a train of tone bursts presented binaurally through headphones. The standard stimuli had a tonal frequency of 650 Hz (60 dB, 200 ms) and occurred with a tonal frequency of 80%. The target stimuli had a tonal frequency of 1 kHz (60 dB, 200 ms) and occurred with a probability of 20%. All participants were informed to pay attention and mentally count infrequent target tones. Interstimulus intervals varied randomly between 1250 and 3000 ms. The N100 latency range was determined to be 65–135 ms, and the P300 latency range was determined to be 280–375 ms. Both the latency and maximum amplitudes were measured for N100 and P300 deflections. Any peaks outside of this range were measured manually, and all peaks were visually examined prior to measurement.

### 2.5. Computerized Assessment Battery Test

The computerized assessment battery test was modified from the CDR computerized assessment battery test used in hundreds of European and North American drug trials which have been previously reported to be sensitive to acute cognitive improvements as well as impairments with a wide variety of substances [11, 12]. Presentation was performed using notebook computers with a high-resolution VGA colour monitor, and, with the exception of written word recall tests, all responses were recorded via a two-button (yes/no) response box. The entire selection of tasks took approximately 20 min. Tests were administered in the following order: word presentation, picture presentation, simple reaction time, digit vigilance task, choice reaction time, spatial working memory, numeric working memory, delayed word recognition, and delayed picture recognition.

**Word Presentation**

Fifteen words, matched for frequency and concreteness, were presented in sequence on the monitor for the participant to remember. The stimulus duration was 1 s, as was the interstimulus interval.

**Picture Presentation**

A series of 20 photographic images was presented on the monitor at the rate of 1 every 3 s, with a stimulus duration of 1 s, for the participant to remember.

**Simple Reaction Time**

The participant was instructed to press the “yes” response button as quickly as possible every time the word “yes” was presented on the monitor. Fifty stimuli were presented with an interstimulus interval that varied randomly between 1 and 3.5 s. Reaction times were recorded in milliseconds.

**Digit Vigilance Task**

A target digit was randomly selected and constantly displayed to the right of the monitor screen. A series of digits was presented in the centre of the screen at the rate of 80/min, and the participant was required to press the “yes” button as quickly as possible every time the digit in the series matched the target digit. The task lasted 1 min and there were 15 stimulus-target matches. Task measures were accuracy (%), reaction time (milliseconds), and false alarms.

**Choice Reaction Time**

Either the word “no” or the word “yes” was presented on the monitor, and the participant was required to press the corresponding button as quickly as possible. There were 50 trials of which the stimulus word was chosen randomly with equal probability, with a randomly varying interstimulus interval between 1 and 3.5 s. Reaction times (millisecond) and accuracy (%) were recorded.

**Spatial Working Memory**

A pictorial representation of a house was presented on the screen with four of its nine windows lit. The participant was instructed to memorize the position of the illuminated windows. In 36 subsequent presentations of the house, one of the windows was illuminated, and the participant decided whether or not this matched one of the lighted windows in the original presentation. The participant made their response by pressing the “yes” or “no” response button as quickly as possible. Mean reaction times were measured in milliseconds, and the accuracy of responses to both original and novel (distractor) stimuli was recorded as percentages used to derive a “percentage greater than chance performance” score.

**Numeric Working Memory**

Five digits were presented sequentially for the participant to hold in memory. This was followed by a series of 30 probe digits for each of which the participant decided whether or not it had been in the original series and pressed the “yes” or “no” response button as appropriate and as quickly as possible. This was repeated two further times with different stimuli and probe digits. Mean reaction times were measured in milliseconds, and the accuracy of responses to both original and novel (distractor) stimuli was recorded as percentages that were used to derive a “percentage greater than chance performance” score.

**Delayed Word Recognition**

The original words and 15 distractor words were presented one at a time in randomized order. For each word, the participant indicated whether or not she recognized it as being included in the original list of words by pressing the “yes” or “no” button as appropriate and as quickly as possible. Mean reaction times were measured in milliseconds, and the accuracy of responses to both original and novel (distractor) stimuli was recorded as percentages that were used to derive a “percentage greater than chance performance” score.

**Delayed Picture Recognition**

The original pictures and 20 distractor pictures were presented one at a time in a randomized order. For each picture, participants indicated whether or not it was recognized as being from the original series by pressing the “yes” or “no” button as appropriate and as quickly as possible. Mean reaction times were measured in milliseconds, and the accuracy of responses to both original and novel (distractor) stimuli recorded as percentages that were used to derive a “percentage greater than chance performance” score.

To avoid learning effect on computerized battery test, the participants were assessed for working memory with different sets of parallel tests at the same difficulty level.

### 2.6. Statistical Analysis

Comparisons between doses were made using analysis of variance (ANOVA) and followed the recommendations of Keppel [13], with planned comparison being made between the placebo and each of the two active treatments utilizing *t*-tests. Statistical significance was regarded at *P*  value <0.05. In order to reject null hypothesis, the sample size justification and power analysis is considered.

Since the study is the preliminary study of clinical trial phase zero, the study is performed in accordance with the United States Food and Drug Administration's (FDA) 2006 Guidance on Exploratory Investigational New Drug (IND) Studies which suggests that the number of the sample size can be a small number approximately 10–15 per group.

## 3. Results

### 3.1. Demographic Data of Subjects

The baseline demographic data for all participants is presented in Table 1. There were no significant differences found in demographic parameters. Therefore, all subjects successfully met inclusion criteria and did not differ significantly.

### 3.2. Effect of *Zingiber officinale* on Event-Related Potential Components (ERPs)

**Figure 1:**
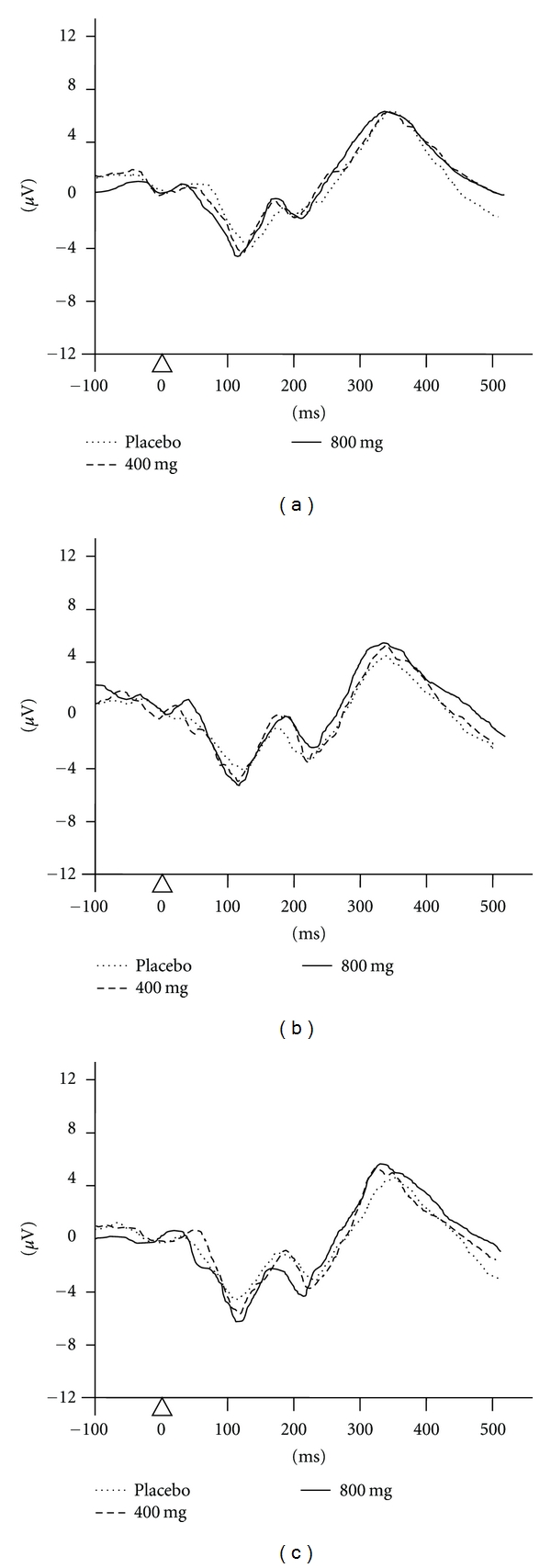
Average waveforms of the auditory event-related-potential at electrode Cz at various periods of treatment; (a) predose baseline, (b) 1st month after substance administration, and (c) 2nd month after substance administration.

The grand average mean for all three conditions is shown in Table 2 and the average waveforms are shown in Figure 1. The predose baseline data of latency and amplitude for both the N100 and P300 of each group showed no significant difference (*F*(2,57) = 0.3765, *P* = 0.6879; *F*(2,57) = 0.1865, *P* = 0.8303; *F*(2,57) = 0.0408, *P* = 0.9600 and *F*(2,57) = 0.0138, *P* = 0.9863, resp.). After one month of treatment, the subjects who received *Zingiber officinale *at a dose of 800 mg showed a significant increase in N100 amplitude (*t* = 3.3076, *P* = 0.0010). After two months, participants who were given *Zingiber officinale* at doses of 400 and 800 mg showed a significant increase in P300 amplitude (*t* = 2.4551, *P* = 0.0094 and *t* = 3.0716, *P* = 0.0020, resp.). Furthermore, subjects who received *Zinigber officinale* at a dose of 800 mg showed a significant increase in N100 amplitude and decreased P300 latency (*t* = 3.1847, *P* = 0.0014 and *t* = 3.6561, *P* = 0.0004, resp.).

### 3.3. Effect of *Zingiber officinale* on Working Memory

Prior to the determination of *Zingiber officinale *on working memory, baseline data and mean predose raw baseline scores for all three conditions (placebo, 400, and 800 mg *Zingiber officinale*) for each individual task scores were subjected to a one-way ANOVA. No significant changes in any parameters were observed.

The mean raw baseline scores and changes from baseline factor scores for each condition across each session are presented in Table 3. It was found that participants who consumed *Zingiber officinale* at the dose of 800 mg/day for one month showed a significant increase in % accuracy of choice reaction time and numeric working memory (*t* = 4.1014, *P* = 0.0001, *t* = 1.9467, *P* = 0.0295, resp.). At two months of intervention, subjects who received *Zingiber officinale* at the dose of 400 mg/day showed a significantly decreased reaction time for word recognition (msec.) (*t* = 2.4000, *P* = 0.0107) while subjects who received *Zingiber officinale* at the dose of 800 mg/day showed significant changes in % accuracy of delayed word recognition, digit vigilance, choice reaction, numeric working memory and spatial working memory (*t* = 2.8799, *P* = 0.0033; *t* = 2.0904, *P* = 0.0217; *t* = 4.2279, *P* < 0.0001; *t* = 2.9313, *P* = 0.0028 and *t* = 3.0325, *P* = 0.0022, resp.). In addition, significant differences in numerous parameters including reaction time of the following: word recognition (*t* = 2.8204, *P* = 0.0037) and choice reaction time (*t* = 2.1778, *P* = 0.0178) were also observed in subjects who consumed *Zingiber officinale* at the dose of 800 mg/day. Therefore, the current data suggests that the plant extract at doses used in this study especially *Zingiber officinale* at the dose of 800 mg/day could improve working memory in all domains including (1) power of attention (obtained from reaction times of simple reaction time, choice reaction time, and digit vigilance tests), (2) the continuity of attention or accuracy of attention (indicated by the elevation of % accuracy of the parameters mentioned above), (3) the speed of memory (indicated by the reaction time of simple reaction, digit vigilance, choice reaction, numeric working memory, picture recognition, and spatial working memory), and (4) quality of memory (indicated by the % accuracy of the parameters mentioned in 3). All participants completed the trial for the whole period. Moreover, no adverse effects after substance administration were observed.

## 4. Discussion

The present study clearly demonstrates that *Zingiber officinale* may enhance both the attention and cognitive processing in middle-aged women. Our event-related potential and computerized battery test (for assessing working memory) data showed that the improvement of cognitive function was observed in all attention and cognitive processing domains. During the last decade, numerous lines of evidence point out that event-related potential (ERP) components are sensitive to the attention and working memory demand of a task [14, 15]. Previous studies show that stimuli that require active discrimination between classes of events typically evoke a large positive voltage deflection in the interval between 300 to 500 ms following the stimulus onset, which is known as P3 or P300 [16]. This component corresponds to mental processes such as recognition, categorization of stimuli, expectancy, or short-term memory. The amplitude of this wave is correlated with individual differences in working memory capacity [17]. P300 latency is regarded as a measurement of relative timing of the stimulus valuation process, indicating stimulus evaluating time [18]. Numerous brain regions including the temporal lobe, parietal lobe, and hippocampus have been proposed to be involved in its generation [19]. Recent findings show that the N100 reflects the process of attention activation, analysis of information based on the physical characteristics of sound, and the formation of memory trace with oscillators in the auditory cortex, prefrontal cortex, hippocampus, and cingulate cortex [20]. Moreover, the amplitude of N100 was also reported to be associated with enhanced memory performance [21], attention [22], expectancy [23], and tasks involving short-term memory [24].

Several studies have also suggested that the high power of attention represents the intensity of concentration at a particular moment, with faster responses reflecting a higher focus of attention. It has been reported that the power of attention can be evaluated in choice reaction time and digit vigilance tests while the continuity of attention is also able to be evaluated using the % accuracy tests mentioned earlier. In addition, the speed and quality of memory are also evaluated by using the reaction time and % accuracy of numeric working memory, spatial memory, and word/picture recognition [25].

With regard to the assessment of working memory via the computerized battery test in accompany with the assessment of brain activity during cognitive performance, our results show that *Zingiber officinale* could enhance both attention and the efficiency of cognitive processing. However, the alteration in attention appears to be more sensitive to the effect of the plant extract rather than the cognitive processing. Previous studies report that the lateral prefrontal cortex (PFC) plays an important role in executive function such as planning, regulating behavior, and finding solutions to novel problems. Moreover, this area also contributes to the significant role of numeric working memory and is also critical for picture and word recognition process [26, 27]. Recent findings also suggest an important role of the hippocampus in spatial working memory [28]. In addition, it was found that dopamine, and norepinephrine play a key role in numeric working memory including word and picture recognition (organized by the lateral PFC), while acetylcholine and serotonin in the hippocampus simultaneously were activated during spatial working memory tasks [29].

*Zingiber officinale* was previously reported to enhance the level of norepinephrine, epinephrine, dopamine and serotonin contents in the cerebral cortex and hippocampus [30]. Moreover, this plant extract and its active component, 6-gingerol, also inhibited the cholinesterase activity which in turn increased acetylcholine (ACh), a neurotransmitter that plays an important role in learning and memory [31]. A recent study demonstrated that ginger extract enhanced the memory performance induced by cerebral ischemia by decreasing infarct volume in both cortical and subcortical areas [6]. Therefore, taking all data together, we suggest that the cognitive enhancing effects of *Zingiber officinale* might be partly associated with the modulation effect of this plant extract on the alteration of both the monoamine system and the cholinergic system in various brain areas, including the prefrontal cortex and hippocampus.

Recent accumulating lines of evidence show that antioxidants could also improve cognitive performance in healthy elderly subjects [32, 33]; therefore, the association between the antioxidant effects of *Zingiber officinale* and the cognitive enhancing effects still cannot be excluded [6, 34].

According to the cognitive enhancing effects of substances possessing antioxidant activity, the concentration of gingerol and shogaol of the extract, and the antioxidant activity of *Zingiber officinale*, we suggest that the cognitive enhancing effect of this plant extract on working memory observed in this study might be partly related to its antioxidant effect. However, the precise underlying mechanism and possible active ingredient responsible for the cognitive enhancing effect of *Zingiber officinale* still require further investigation.

Although the side effect of *Z*. *officinale* extract is rare which is corresponding with our data [35–37], some minor adverse effects at higher doses such as gastrointestinal disturbance, sleepiness, restlessness, sedation, and heartburn were also reported [38–40]. Moreover, the extract could therefore also possibly interact with medications including anesthesia, anticoagulants, and analgesics leading to arrhythmias, poor wound healing, bleeding, photosensitivity reaction, and prolonged sedation [41, 42]. Therefore, the application in the mentioned conditions should be performed with caution.

## 5. Conclusion

**Figure 2:**
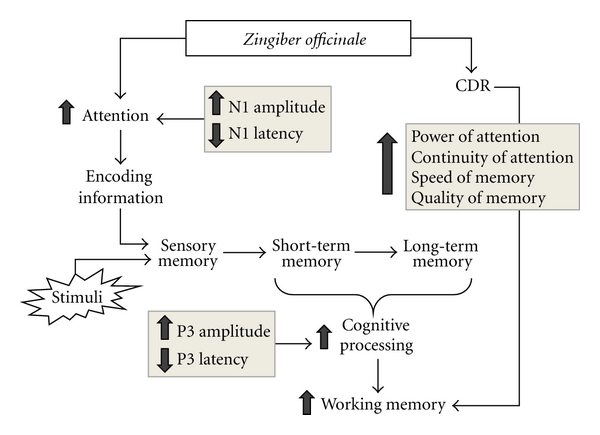
Effect of *Zingiber officinale* on attention, cognitive processing capabilities, and working memory of healthy, middle-aged women.

The present study demonstrates that ginger extract enhances both attention and cognitive processing capabilities of healthy, middle-aged women, with no side effects reported. Therefore, our data reveal that *Zingiber officinale* extract is a potential brain tonic to enhance cognitive function for middle-age women (Figure 2). However, further study about the precise underlying mechanism especially the effect of the extract on the alteration of acetylcholine and monoamine transmitters should be performed.

## Figures and Tables

**Table 1:** Demographic data of subjects (*n* = 20/group).

Baseline data	Placebo	400 mg	800 mg
Age (years)	53.92 ± 3.82	54.33 ± 4.12	54.33 ± 3.17
Education (years)	5.50 ± 3.70	5.40 ± 3.68	5.15 ± 2.74
Full scale IQ	98.95 ± 4.42	99.75 ± 4.23	98.85 ± 6.01
Blood sugar	90.06 ± 8.45	89.10 ± 13.08	91.15 ± 10.16
Body mass index	21.95 ± 1.90	22.78 ± 2.06	23.12 ± 1.83
Blood pressure systolic (mmHg)	121.00 ± 7.72	117.70 ± 8.49	117.85 ± 9.76
Blood pressure diastolic (mmHg)	82.25 ± 2.53	82.80 ± 2.73	80.50 ± 3.73
Menstrual cessation (years)	3.95 ± 1.60	3.75 ± 1.48	4.05 ± 1.57

Data were presented as mean ± SD. *P* and *F* values were compared between groups.

**Table 2:** Effect of *Zingiber officinale* on auditory event-related potential.

Wave	Predose baseline score		Postdose score	
			1 month	2 months
N100 latency	Placebo	116.80 ± 1.385	114.50 ± 11.97	113.85 ± 10.24
	400 mg	114.35 ± 11.44	113.25 ± 11.85	110.35 ± 10.17
	800 mg	114.05 ± 8.31	109.95 ± 8.72	106.75 ± 9.13

N100 amplitude	Placebo	5.70 ± 10.08	5.65 ± 1.08	5.70 ± 1.07
	400 mg	5.90 ± 1.37	6.40 ± 1.18	6.55 ± 1.05
	800 mg	5.75 ± 1.29	7.05 ± 1.19**	6.90 ± 0.96***

P300 latency	Placebo	332.70 ± 12.96	330.30 ± 11.02	332.35 ± 8.99
	400 mg	332.25 ± 13.81	329.45 ± 11.78	323.85 ± 13.10
	800 mg	332.90 + 10.20	325.60 ± 12.91	321.35 ± 9.77***

P300 amplitude	Placebo	7.25 ± 1.10	7.25 ± 1.06	7.20 ± 1.05
	400 mg	7.25 ± 1.01	7.50 ± 1.23	8.10 ± 1.16**
	800 mg	7.20 ± 1.10	7.90 ± 1.02	8.40 ± 1.35**

The amplitudes and latencies of event-related potential elicited by oddball paradigm at Cz electrode were measured. Data are presented as mean ±SD (*n* = 20/group).

**, ***P value < 0.05, 0.01, and 0.001 compared to placebo-treated group, respectively.

**Table 3:** Effect of *Zingiber officinale* on working memory assessing via computerized battery test.

Measurement	Predose baseline score		Post-dose score	
			1 month	2 months
(1) Delay word recognition (% accuracy)	Placebo	72.99 ± 8.97	73.88 ± 9.25	75.33 ± 8.94
	400 mg	74.83 ± 8.27	75.83 ± 8.58	80.17 ± 7.45
	800 mg	74.83 ± 13.39	79.00 ± 12.14	84.89 ± 8.03**

(2) Delay word recognition reaction time (msec.)	Placebo	1242.56 ± 217.14	1247.75 ± 256.32	1245.06 ± 165.89
	400 mg	1226.06 ± 161.82	1221.45 ± 176.35	1120.67 ± 111.2*
	800 mg	1261.14 ± 176.75	1109.55 ± 171.69	1099.67 ± 185.22**

(3) Simple reaction time (msec.)	Placebo	619.05 ± 222.35	622.50 ± 175.01	625.15 ± 161.96
	400 mg	616.25 ± 195.39	611.95 ± 185.75	596.30 ± 126.60
	800 mg	623.25 ± 191.43	614.30 ± 175.48	573.95 ± 177.20

(4) Digit vigilance (% accuracy)	Placebo	43.35 ± 6.84	42.90 ± 7.95	42.45 ± 8.744
	400 mg	42.90 ± 5.05	43.45 ± 9.93	43.70 ± 6.52
	800 mg	44.75 ± 5.63	44.65 ± 6.45	48.40 ± 5.40*

(5) Digit Vigilance reaction time (msec.)	Placebo	631.65 ± 140.92	622.25 ± 109.91	626.60 ± 122.24
	400 mg	620.00 ± 122.74	621.80 ± 105.69	594.70 ± 83.15
	800 mg	623.75 ± 109.55	608.70 ± 130.34	587.40 ± 71.65

(6) Digit vigilance false alar number	Placebo	8.85 ± 2.39	8.7 ± 1.55	8.5 ± 1.35
	400 mg	8.85 ± 2.18	8.25 ± 1.61	8.05 ± 1.43
	800 mg	8.65 ± 2.13	7.35 ± 1.34	7.1 ± 1.44**

(7) Choice reaction time (% accuracy)	Placebo	79.90 ± 7.40	81.70 ± 6.68	80.55 ± 7.47
	400 mg	80.00 ± 8.86	84.95 ± 9.23	85.40 ± 7.92
	800 mg	79.05 ± 8.53	89.95 ± 8.26**	90.00 ± 7.82***

(8) Choice reaction time response (msec.)	Placebo	976.00 ± 168.70	964.25 ± 100.98	961.30 ± 135.76
	400 mg	964.55 ± 191.10	944.80 ± 128.93	912.10 ± 71.58
	800 mg	980.35 ± 197.24	915.90 ± 72.00	874.65 ± 50.59*

(9) Numeric working memory (% accuracy)	Placebo	73.90 ± 10.40	75.00 ± 10.43	74.70 ± 10.54
	400 mg	75.50 ± 8.67	77.10 ± 10.95	81.35 ± 9.57*
	800 mg	76.45 ± 9.69	82.40 ± 9.63*	85.00 ± 8.72**

(10) Numeric working memory reaction time (msec.)	Placebo	1334.50 ± 226.25	1348.29 ± 209.25	1335.70 ± 203.13
	400 mg	1339.40 ± 234.61	1343.90 ± 236.38	1325.05 ± 171.35
	800 mg	1335.60 ± 260.95	1337.10 ± 170.24	1313.95 ± 138.71

(11) Picture recognition (% accuracy)	Placebo	72.99 ± 8.97	73.88 ± 9.25	75.33 ± 8.94
	400 mg	74.83 ± 8.27	75.83 ± 8.58	80.17 ± 7.45
	800 mg	74.83 ± 13.39	79.00 ± 12.14	84.89 ± 8.03

(12) Picture recognition reaction time (msec.)	Placebo	1256.88 ± 239.51	1247.75 ± 156.32	1245.06 ± 165.89
	400 mg	1224.88 ± 185.08	1221.45 ± 176.35	1120.67 ± 111.25
	800 mg	1234.61 ± 197.52	1109.55 ± 171.96	1099.67 ± 185.22

(13) Spatial working memory (% accuracy)	Placebo	66.25 ± 6.64	66.13 ± 5.39	66.29 ± 4.59
	400 mg	66.11 ± 5.47	66.95 ± 5.47	70.53 ± 5.34**
	800 mg	66.33 ± 6.88	68.39 ± 7.08	71.77 ± 4.12**

(14) Spatial working memory reaction time (msec.)	Placebo	1799.25 ± 33.45	1817.30 ± 203.17	1844.10 ± 232.15
	400 mg	1784.30 ± 191.92	1707.85 ± 296.62	1761.55 ± 165.96
	800 mg	1712.75 ± 219.45	1695.75 ± 200.13	1704.40 ± 309.16

Subjects were measured for power of attention, continuity of attention, speed of memory, and quality of memory by using computerized battery test. Data are presented as mean ± SD (*n* = 20/group).

*, **, ***P value < 0.05, 0.01, and 0.001 compared to placebo-treated group, respectively.
